# Breeding better crops: lessons from the homologous recombination pathway

**DOI:** 10.1007/s00122-026-05297-4

**Published:** 2026-07-24

**Authors:** Anastasia Kolesnikova, Andrew Armitage, Klara Hajdu, Helen Cockerton

**Affiliations:** 1 University of Kent, Giles Lane, Canterbury, Kent, CT2 7NZ UK; 2 Southampton University, University Road Southampton, Southampton, SO17 1BJ UK; 3 Natural Resources Institute (NRI), University of Greenwich, Central Avenue, Chatham Maritime, Kent, ME4 4TB UK; 4 Wye Hops Ltd., China Farm, Harbledown, Canterbury, Kent, CT2 9AR UK

## Abstract

Homologous recombination is a key feature of sexual reproduction, allowing reciprocal exchange of parental chromosome segments. The resulting chromosomal crossovers lead to the merging of DNA segments inherited from different parents, allowing for the accumulation of unique combinations of genetic information on a single molecule. The number of DNA crossover events is tightly controlled and relatively restricted in many organisms, including plants. However, increasing the number of crossover events should theoretically generate a greater number of new phenotypes, some of which may prove beneficial in new environments. Crop breeders, whether intentionally or not, are developing new varieties within the confines of crossover numbers. As such, work describing the mechanism of homologous recombination may provide greater insight into how the process can be modified to enable optimal crop improvement. Here, we provide a comprehensive, up-to-date protein network of homologous recombination processes in plants. It is anticipated that this network will assist in the understanding of plant DNA crossovers and chromosomal segregation during meiosis. In addition to providing this compilation of current understanding, we also assess the extent to which homologous recombination is being altered by breeders, discuss new technologies that could be used to manipulate crossover frequencies and detail research highlighting the potential benefits of embracing this knowledge.

**Supplementary Information:**

The online version contains supplementary material available at 10.1007/s00122-026-05297-4.

## Introduction

Meiosis is a process whereby a single cell undergoes two rounds of division leading to the generation of four genetically distinct sex cells or gametes. The resulting gametes contain only half of the genetic material required to make a new individual. As such, to make a complete individual, two gametes must fuse either through inbreeding (fusing with their own gamete) or outcrossing (fusing with a gamete of another individual). Novel DNA combinations are generated through two processes: meiosis and outcrossing. Meiosis leads to variation in genetic combinations through two mechanisms: firstly, through the random independent assortment of the maternal and paternal chromosomes into each gamete; and secondly, through recombination, which is a mechanism that allows genetic exchange between two homologous chromosomes, where crossovers enable new combinations of alleles to arise within a single molecule of DNA (Muller 1916; Gottlieb et al. 2018). It is well-established that these crossovers between chromosome arms in particular are an important component of generating genetic diversity in gametes (Rowan et al. 2019). Ultimately, new combinations of DNA contribute to the ability of a population to adapt in a changing environment, where individuals that contain a favourable complement of DNA have a higher fecundity and contribute to a greater extent in the next generation (Carja et al. 2014). Crossovers are also desirable for plant breeders, as they can generate new combinations of linked genes that are inherited as a distinct unit/single section of DNA. These desirable combinations of linked genes can be selected and fixed within varieties through selection by the breeders themselves. Moreover, crossovers can break up undesirable linkage between genetic components controlling desirable and undesirable traits.

At least one crossover between homologous chromosomes is required during prophase I of meiosis in order to ensure equal segregation of chromosomes and to avoid aneuploidy (Roeder 1990). This is particularly important where a full complement of chromosomes is required to achieve balanced gene dosage in the resulting progeny (Potapova and Gorbsky 2017). These processes are closely associated with DNA repair. Errors in the homologous DNA repair pathway frequently lead to abnormal DNA segregation, whereas errors in the homologous recombination pathway can result in high or low crossover frequencies between chromosomes (Potapova and Gorbsky 2017; Serra et al. 2018). Understanding the pathways underpinning homologous recombination is critical to working out how we can modulate the frequency of crossovers and harness the variation generated by recombination in order to provide crop breeders with new strategies and tools to generate desirable varieties.

**Fig. 1:**
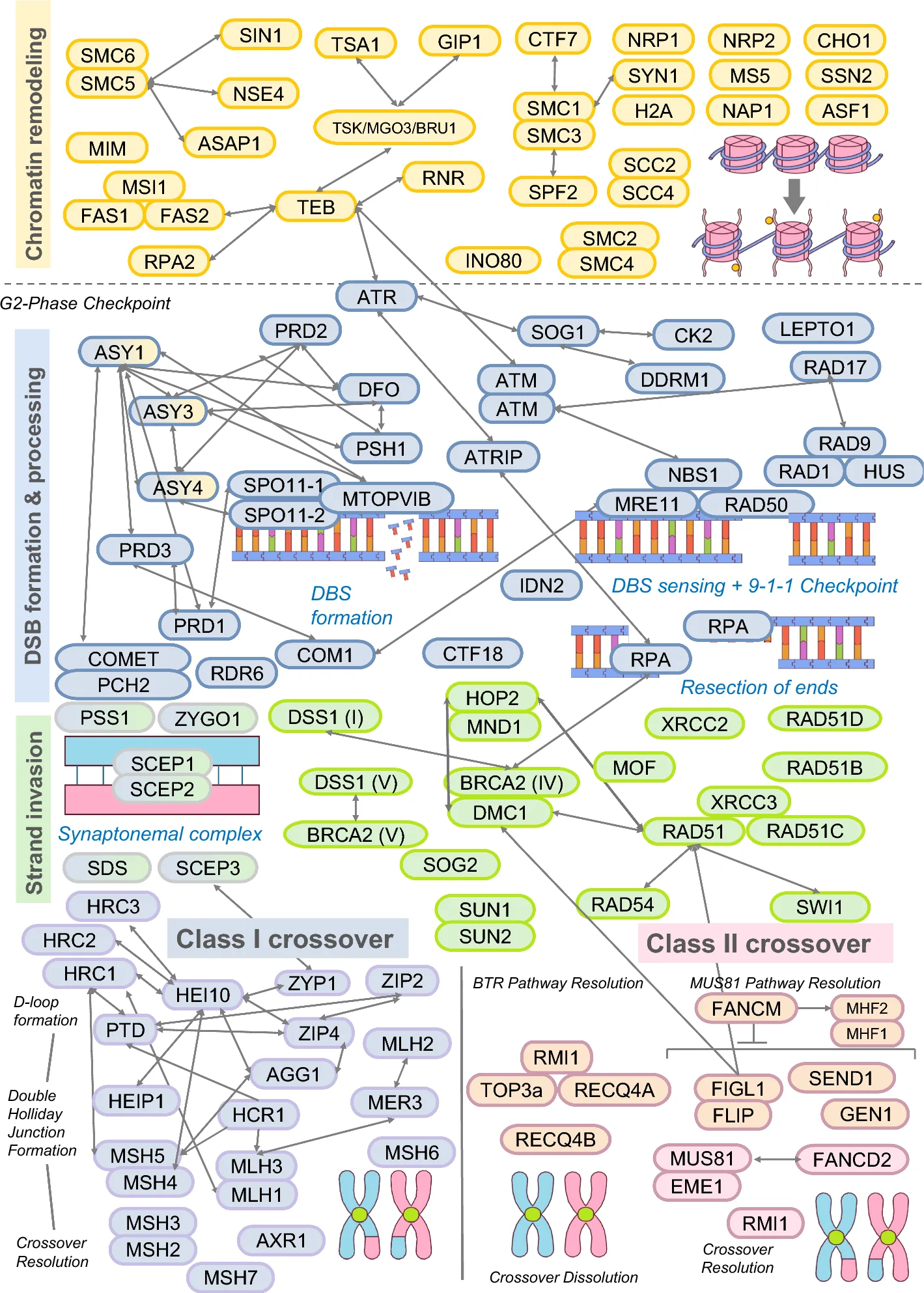
The molecular processes that take place during plant meiosis, with the colour of the boxes and stadiums identifying proteins belonging to each stage. The processes include: chromosomal alignment and chromatin remodelling (yellow); double-strand break (DSB) formation and processing (blue); strand invasion (green); class I crossovers (purple), class II crossovers (pink) and class II associated dissolution (orange). Grey arrows indicate known interactions between proteins. Notations inside stadiums denote proteins. Complexes are represented by connected protein stadiums. For full details on protein names, functions and associated citations, see Supplementary Table 1. Image created with Canva

Extensive work has led to the functional characterisation of genes involved in plant homologous recombination. Here, we build upon in-depth reviews of the meiotic recombination pathway in *Arabidopsis thaliana* (Osman et al. 2011; Lloyd 2023) and synthesise a schematic summary detailing the current understanding of meiotic protein interactions involved in homologous recombination, as reported in *A. thaliana* and wider plant species (Fig. 1; Supp. Table 1).

## Overview of crossover formation

Before the DNA is divided between the two daughter cells, crossovers occur between the maternal and paternal version of each chromosome. First, chromosomes duplicate to double the amount of DNA present in the cell during the synthesis (S) phase of interphase. After duplication, the homologous chromosomes align precisely in pairs during prophase of meiosis I and a double-strand break forms in one chromosome. The two chromosomes then become physically joined to one another in the region of the break, through the formation of a synaptonemal complex. The physical joining of the broken DNA ends from each homologous chromosome occurs through a process called strand invasion. Strand invasion can lead to two types of crossovers that are regulated by two independent pathways: Class I and Class II. Where crossover formation is unsuccessful, no crossover occurs, whereby DNA breaks are repaired to restore the chromosome to the original unbroken state. DNA repair resulting in no crossing over occurs by one of the four pathways, each of which are controlled by a set of core proteins (or protein homologues), that conduct the same function across the different pathways (Lloyd 2023). Greater detail of the proteins involved in each step of the homologous recombination pathway is provided in Fig. 1, and references are provided in Supp. Table 1.

### Chromosome alignment and remodelling

The condensation and alignment of sister chromatids is mediated by multiple protein complexes, including the Structural Maintenance of Chromosomes (SMC) complexes that hold the two sister chromatids together, stimulating the start of the homologous recombination process (Lehmann 2005; Kegel and Sjögren 2010). During chromatid alignment, a distinct complement of proteins play a role in chromatin remodelling, whereby chromatin is unravelled from its condensed state into an accessible form to allow the DNA to “break” and enable repair machinery to access the potential crossover site (Rosa et al. 2013). Alongside chromatin remodelling, the proteinaceous chromosome axis forms (ASY1 and ASY3) along the length of each sister chromosome with loops of chromatin extending from the backbone (Lloyd 2023).

### Breaking of DNA

After axis formation and chromatin remodelling, double-strand breaks form with the help of the SPORULATION 11 (SPO11) protein complex (Stacey et al. 2006). The SPO11 heterodimer mediates and catalyses the formation of meiotic double-strand breaks; after attachment, DNA is then cleaved either side of the SPO11 proteins. The MRN complex (MRE11-RAD50-NBS1) then releases SPO11 attached to the 5’ DNA break site resulting in “jagged” asymmetrical exposed DNA. This MRN complex also activates the protein kinase (ATM) that relays the signal that DNA damage has occurred (Sharma et al. 2021). This sets off a signalling cascade through monomerisation of ATM kinase (an otherwise catalytically inactive dimer) which not only recruits gatekeeper proteins (RPA) to protect the single-strand DNA overhangs, suppressing the recombinase activity but also activates multiple checkpoint proteins (RAD17 and the 9-1-1 checkpoint complex: RAD9, RAD1 and HUS) which, in turn, act to prevent joining between strands from the original chromosome (via alt NHEJ) (Heitzeberg et al. 2004; Harper and Elledge 2007; Osman et al. 2011; Tsai and Kai 2014; Aklilu et al. 2014; Kurzbauer et al. 2021).

After double-strand break formation, a synaptonemal complex forms along the entire length of the two chromosome axes binding the two homologous chromosomes together. Transverse filament proteins (ZYP1) bind the two axial elements together like a zipper (Higgins et al. 2005). When researchers have deleted these ZYP1 zipper proteins, they discovered that the synaptonemal complex was required to both limit the crossover frequency and maintain heterochiasmy (whereby male meiocytes experience greater crossover frequencies than female meiocytes) (Capilla-Pérez et al. 2021).

### To cross or not to cross?

DNA repair leading to crossover formation between homologous chromosome arms can occur by one of the two pathways: the ZMM pathway, which results in a Class I crossover (Dluzewska et al. 2018) or the MUS81 pathway, which results in a Class II crossover (Hartung et al. 2006). As part of the MUS81 pathway, dissolution of Holliday junctions can occur through the BTR complex (*RMI1, TOP3* and *RECQ4A* (plant homologue of yeast *BLM*)) (Olivier et al. 2016). The location and number of Class I crossovers is tightly controlled to ensure that multiple crossovers do not occur within the same region; by contrast, Class II crossovers may occur next to other crossovers and are not sensitive to interference from neighbouring crossovers (Ziolkowski 2023; Mercier et al. 2005). However, dissolution of the MUS81 pathway can occur via the BTR complex leading to the rejoining of the original chromosome arm, and thus, this repair results in no crossover. Similarly, four additional pathways also lead to the rejoining of the original chromosome arm resulting in no crossover (Classical Non-Homologue End Joining, Alternative Non-Homologue End Joining, Alternative End Joining Pathway, Single-Strand Annealing, or Sequence-Dependent Strand Annealing). A repair leading to no crossover is far more likely than a crossover event with only 6.7% of synaptic complexes leading to the formation of a crossover (Sanchez-Moran et al. 2007). Indeed, in Arabidopsis, around 150–250 double-strand breaks are formed across the genome, but only around 10 become crossovers (Mercier et al. 2015). Similar patterns are found in economically important crops; from the nearly 500 original breaks in maize, leading to around 20 crossovers (Dluzewska et al. 2018) and 40–50 crossovers observed in wheat (Gardiner et al. 2019).

Whether or not a crossover occurs is tightly regulated by a series of interacting proteins acting as check points to permit crossover formation. As mentioned above, the signalling cascade controlled by ATM kinase is a key regulator that not only relays the DNA damage signal and mediates the response to prevent recombining of homologous chromosome arms but also plays a role in limiting the number of double-strand breaks formed through mediating chromatin loop size and influencing the length and width of synaptonemal complexes (Kurzbauer et al. 2021). Whether or not a crossover is formed and is determined at the strand exchange step and exactly how this is regulated remains unknown.

### Homologous recombination

Strand invasion is the process by which DNA on the intact homologous chromosome arm that matches the broken strand is identified and amplified. Strand invasion is facilitated through DSS1 (I), which searches for and identifies a region of homology in intact duplex DNA. This mechanism promotes homology directed repair allowing precise repair of double-strand breaks using the sister chromatids as a template, a complex process mediated via multiple proteins. Following strand invasion, a strand from the homologous chromosome forms a displacement-loop (D-loop) to provide a template for the repair of the double-strand break (catalysed by the recombinases DMC1 and RAD51) (Singh et al. 2017). From here, a recombination intermediate structure is formed, termed a double Holliday junction; these junctions can be resolved through crossover formation or dissolution leading to no crossover.

### Pathway conservation

Details of the plant homologous recombination pathway are predominantly derived through work conducted on the model organism *Arabidopsis thaliana* and in the monocot crop rice (*Oryza sativa*) (Whitbread et al. 2021). As such, additional work is required to understand the conservation of gene function across the wider plant kingdom. Indeed, certain proteins involved in homologous recombination processes have been shown to function slightly differently across different plant species (Whitbread et al. 2021). For example, it is known that tomato has different uses for the proteins that make up the BTR complex; whereas mutation of the *FIGL1* gene led to sterility in pea, rice and tomato, but not Arabidopsis (Mieulet et al. 2018; Whitbread et al. 2021). Furthermore, several genes have been shown to be required for double-strand break formation in rice but not Arabidopsis (Wang et al. 2021). As such, relative conservation of homologous recombination gene function across the plant kingdom will require extensive investigation. By contrast, *HEI10, RECQ4A, RAD17* and the 9-1-1 complex have essential roles that have been maintained across all the eukaryotes (Heitzeberg et al. 2004; Hartung et al. 2006; Chelysheva et al. 2012; Lim 2015). Genetic manipulation of genes with conserved functions may produce similar results across multiple crop species and hence serve as attractive options for such manipulation.

### Boosting recombination frequency

As part of crop breeding, it is important to understand the processes that lead to the generation of variation and the co-inheritance of alleles of interest, as modulating these processes underpins our ability to utilise the genetic and resulting phenotypic variation that they generate. High recombination frequencies can lead to breaking up beneficial haplotypes or links between complementary genes, and so, high recombination is not typically advantageous in natural populations (Otto and Lenormand 2002). The locations where crossovers form are typically in non-random regions of the genome termed “hotspots”. However, the “hotspot paradox” means that mutations that eliminate hotspot activity are likely to become fixed in the population (Dluzewska et al. 2018), and thus, high crossover frequency is selected against. However, recombination is a beneficial process for populations under temporal/spatial variable selection pressures (Otto 2009). Such selection pressures are analogous to scenarios where breeders apply artificial selection to populations for good performance in a new environment or towards a desirable phenotype. It follows that creating genotypically diverse individuals through elevating recombination rates can be seen as a desirable strategy for crop breeders. As such, harnessing “rapid evolution” individuals through modulating the regulation of genes that control crossover frequency has the potential to assist the crop development community in generating better crops faster.

Multiple studies have identified the factors controlling recombination frequencies in plants (Serra et al. 2018). This knowledge has facilitated upregulation and knockout of meiotic genes leading to high recombination frequencies in mutant gametes. Some meiotic genes have proven to be good universal targets where they are relatively conserved across the plant kingdom. Indeed, knocking out the function of *RECQ4A* in rice, pea and tomato has been shown to boost the number of crossovers occurring during meiosis by a factor of three, whilst targeting the anti-crossover helicase FANCM in pea and rice led to a twofold crossover increase (Crismani and Mercier 2012; Mieulet et al. 2018). Additional targets for manipulation are genes that regulate crossover number or mediate crossover interference. Two such targets, HEI10 and ZYP1, are not only heavily involved in regulating crossover numbers but they also modulate the intensity of crossover interference to influence the space between crossovers (France et al. 2021; Morgan et al. 2021; Lloyd 2023).

A number of studies have strived to influence crossover frequency and location through introducing abiotic stresses from temperature, UV radiation and precipitation. The “eco-crossover” hypothesis suggests populations under stress should modify crossover frequency to generate non-random diversity to adapt in a timely manner (Olovnikov 2022). These attempts have been found to produce highly variable results. For example, high temperatures have been reported to both impair synapsis and lower crossing over, but also lead to hyper-recombination in *A. thaliana* (Modliszewski et al. 2018; De Storme and Geelen 2020). Meiotic recombination may be compromised in polyploid Arabidopsis at high temperatures, with similar inhibitory effects reported in polyploid rice and canola but not within naturally hexaploid wheat (Fu et al. 2022). Beyond temperature impacts, plant nutrition may play an important role as high magnesium levels have been demonstrated to increase meiotic crossover frequencies in wheat (Rey et al. 2018). An increased understanding of the connection between the abiotic stress response and crossover formation provides an opportunity to identify indirect targets to facilitate artificial increases in crossovers. In addition, modifying crossover rates using environmental stressors may lead to the appearance of improved phenotypes specific to the stressor; therefore, breeding to address climate adaptations for certain crops may benefit from such an approach.

### Beyond recombination: Apomeiosis and the fixation of heterosis

Meiosis is not always a desirable occurrence in crop production. Although meiosis can help to combine beneficial traits as part of varietal development, once the optimum phenotype has been generated, continued recombination can break apart desirable combinations. Apomixis is a process by which seeds that are identical to the mother plant can be generated without fertilisation or meiosis occurring (Goeckeritz et al. 2024). Staple crops do not typically undergo this asexual reproduction strategy in their seeds. Many staple crop breeders embrace a phenomenon known as hybrid vigour (or heterosis) to generate varieties that outperform the parents. Heterosis can result in increased yield, vigour, tolerance to stress and uniformity of crop for easier machine harvesting, and has been applied to ~ 50 crop species, including maize (Hochholdinger and Yu 2025). Unfortunately, the F2 progeny of these high-performing hybrids are highly variable and are undesirable for cultivation. Therefore, to retain the benefits of heterosis, crop producers must repeat the original cross. The ability to generate seed identical to the hybrid parents stands to have a large impact on the ease with which we can harness sustained hybrid vigour (Fiaz et al. 2021). Apomixis occurs naturally in several crop species, for example in papaya (Vegas et al. 2003); it can also occur in cassava alongside polyploidisation, although the frequency is relatively rare. For cassava, apomixis occurs by a completely unique mechanism whereby somatic embryos arise inside the seed coat itself (Freitas and Nassar 2013). Apomixis can be induced through multiple strategies. Experimentally, apomixis has been induced in *A. thaliana* through mutation of *MiMe*, a gene system consisting of *SPO11-1*, *REC8* and *OSD1*, which was found to turn the process of meiosis into mitosis when mutated. In *MiMe* mutants, megasporocytes skip the second phase of meiosis to produce seeds genetically identical to the parents (d’Erfurth et al. 2009). *MiMe* has also been successfully targeted in rice, a genetically distant monocot (Mieulet et al. 2016) and elite hybrid tomatoes (Wang et al. 2026), leading to staple crops with the ability to produce asexual seeds. Researchers have also demonstrated the potential of targeting *MiMe* where clonal seed could be produced in sterile polyploid tomatoes (Wang et al. 2024). Another strategy to achieve apomixis involves targeting the *BABYBOOM1* gene, which in elite hybrid rice lines results in seed parthenogenesis whilst the capacity for meiosis is retained, with > 95% of the resulting seeds being clonal (Song et al. 2024). Synthetic clonal seed production bypassing sexual reproduction has also been achieved in maize lines (Shi et al. 2025). Existing polyploid crops may stand to benefit substantially from this. Bananas may be a prime candidate for apomictic development, as cultivated desert bananas are triploid and infertile, requiring clonal propagation post initial crossing. As such, apomictic bananas may have an impact on production and germplasm conservation of the species (Lin et al. 2024).

### Manipulating the locations of crossovers

Hotspots for crossover events are typically observed in gene-rich regions, peaking in promoter and terminator regions, with crossovers forming along open chromatin that is nucleosome free and hypomethylated (Choi et al. 2018; Dluzewska et al. 2018; Lloyd 2023; Epstein et al. 2023). Moreover, crossover locations are typically conserved with four out of five crossovers occurring within the same “hot regions”, represented by 26% of the *A. thaliana* genome (Choi et al. 2013). Breeders frequently want to enhance recombination in recalcitrant or “cold regions” of a plant’s genome, thus enabling them to reduce the linkage drag associated with desirable alleles, with new crossovers breaking linkage and expediting the removal of undesirable alleles (Blary and Jenczewski 2019). For example, one of the major pinch points in wheat breeding is the lack of recombination between alien homoeologous chromosomes from crop wild relatives. Indeed, it has been shown that manipulation of the *RECQ4A* gene in wheat was not only able to enhance recombination frequency but also boost recombination between homoeologous chromosomes; the *Ph1* and *Ph2* loci containing genes *ZIP4* and *MSH7,* respectively, have also been used to increase recombination between homoelogues (Bazile et al. 2023). However, others have observed that a constant boost to crossover number between homoeologous chromosomes leads to excessive chromosome rearrangements and thus increased infertility in progeny (Sánchez-Morán et al. 2001). As such, it is clear that transient regulation of meiotic genes is key to achieving the benefits of enhanced crossovers as achieved by Knight et al. (2010). Others have sought to generate hypomethylated mutants in order to boost crossover formations in cold genome locations; however, this has been shown to have pleiotropic impacts and even lethality in the resulting plants (Hu et al. 2014). A more targeted approach to enhance recombination in cold regions of the genome employs CRISPR dead Cas9 technology to allow precise modification of methylation signals in a region of the genome and thus enable precision induction of crossovers (Dluzewska et al. 2018). Moreover, the use of spray-induced gene silencing provides opportunity for transient knockdown of recombination genes for a single generation of sex cell production. This, in turn, prevents the continuous crossing over seen in stable transformants whilst also preventing the breakup of beneficial combinations formed during earlier inductions (Younis et al. 2014; Dluzewska et al. 2018). The manipulation of pro and anti-crossover factors through modification of DNA through gene editing, mRNA foliar application, spray-induced gene silencing, or the targeted manipulation of epigenetic factors to influence the locations of crossovers by breeders is termed “Controlled recombination” (Taagen et al. 2020; Uslu et al. 2025).

### The impact of chromosome architecture on crossovers

Natural variation in the pattern of crossover formation can be seen between different crops whereby the architecture of chromosomes appears to have a strong influence on crossover locations. Smaller genomes tend to have consistent crossover frequencies along the lengths of their chromosomes, whereas large genomes typically possess highly repetitive regions with the majority of crossovers forming in the distal euchromatin regions at the ends of the chromosomes (Wang et al. 2021). For example, in maize with a genome size approximately 18 times the size of Arabidopsis, only 10% of the chromosomal regions undergo crossovers, leaving large interstitial cold regions intact (Wang et al. 2021, Lui et al. 2019, Kiannan et al. 2018). Transposable elements (TEs) can play a large role in plant genome expansion and also impact crossover locations (Sergeeva and Salina 2011). Genomic investigation of cucurbits has identified that genome expansion in melon has led to the formation of two distinct genome compartments that are subject to processes of genome evolution at different rates. A core suite of ancestrally-shared genes is located in the euchromatin region which allows crossovers, whereas the pericentric region, which lacked crossovers, contained “young” melon-specific genes which were under selection through TE manipulation (Morata et al. 2018). Further investigation into plant genome compartmentalisation may provide a TE-based strategy of creating genetic variation in crop genomes or perhaps offering a solution to target crossover-poor TE-rich regions.

### Exploitation in breeding programmes

Blary and Jenczewski (2019) discuss the trade-off of crossovers between the downside of breaking up beneficial alleles and the upside of creating new variants. They suggest that a boost in crossover rate may be more useful where prebreeders are seeking to introgress wild or non-refined material into their breeding programmes, rather than elite variety generation (Blary and Jenczewski 2019). In effect, crossovers can help remove the unrefined and undesirable genetic material that is inherited alongside that controlling the trait(s) of interest. Ironically, breeders may unconsciously impact recombination frequencies through introgressing wild material into breeding populations. Whilst a boost in adaptive diversity can be achieved through incorporation of wild relatives into elite breeding germplasm, these crosses can also be associated with a decrease in crossover frequency (Bohra et al. 2022). Indeed, crosses between wild and domesticated varieties in tomato have been shown to decrease crossover frequencies (Demirci et al. 2017; Dreissig et al. 2020; Fuentes et al. 2024). This reduction is likely due to the structural differences between the two genomes that have diverged over time. The reduction in crossover frequency associated with introgression of crop wild relatives highlights the importance of developing technologies to boost recombination frequencies.

More generally, it is important to keep making genetic gains within breeding programmes and to embrace new technologies in order to enhance staple crop yields. For example, in wheat, enhancing crossover numbers could lead to greater yield improvements, especially in genomic cold regions that contain a high proportion of genes, such as chromosome 3B of wheat, where 70% of its genes are contained in cold regions (Choulet et al. 2014; Fayos et al. 2022; Epstein et al. 2023). Current literature is in consensus that there is considerable potential for manipulating crossover numbers in breeding programmes whilst acknowledging that there has been limited application of this knowledge to date (Blary and Jenczewski 2019). However, this may change with the advent and development of novel crop modification tools detailed above (Younis et al. 2014; Dluzewska et al. 2018).

### Where next?

The global human population is expected to rise to 9.7 billion by 2050 (Gu et al. 2021). Feeding this growing population is a substantial challenge, particularly when combined with existing food poverty and the need to reduce our land-use footprint. As such, there is a pressing need to improve crop production through development and implementation of novel crop breeding technologies. Neo-domestication breeding approaches such as gene-editing rely more heavily on understanding and manipulating the genomes of species through molecular approaches, as such, there is scope to apply the understanding of crossover formation during the initial phases of such breeding projects (Gutaker et al. 2022). Increasing crossover frequency in populations of interest can help to generate a greater number of phenotype combinations, some of which are likely to contain rare crossover events which would be of interest to breeders (Bernardo 2025). Given that high recombination benefits non-elite germplasm; deployment of these techniques may be of particular benefit to underutilised and orphan crops; locally grown species with limited production and consumption. Such plants are of particular interest as climate resilient crops as they are often adapted to harsh climates, are nutritious, and hold potential for addressing food security. Genome sequencing projects and development of meiotic atlases have now been achieved for many underutilised crops, including lablab, golden thistle and cherimoya; however, there has been limited, if any, progress in increasing recombination levels in these species (Chapman et al. 2022; Talavera et al. 2023; Tek et al. 2025). Existing diverse germplasm collections, such as those at genebanks of the Consultative Group for International Agriculture (CGIAR) research centres, can serve as starting points to screen for inherent differences in recombination efficiency across species or provide valuable genetic variation which can be introduced into elite varieties through recombination technology. In particular, cassava is a prime candidate crop to benefit from recombination enhancement technology. Cassava breeding is in its formative years. Indeed, there is a lack of pre-existing elite germplasm, and limited genetic improvement was made during the green revolution (Ceballos et al. 2004). Notwithstanding this, cassava is a nutrient dense food security crop that can provide reliable nutrition when grown in conditions where other crops would fail, as such, genetic refinement using advanced breeding strategies has the potential to boost yields and further enhance resilience (Amelework et al. 2021). Ultimately, achieving long-term food security requires large-scale coordinated efforts amongst breeders, genbanks, geneticists and other stakeholders across the food chain.

## Conclusion

Here, we provide a summary and visualisation of the current state of knowledge for the molecular interactions controlling homologous recombination in plants. We discuss the parameters within which breeders may be able to make use of this knowledge, and discuss the potential for novel technologies to assist transient manipulation of crossover frequency or genomic location. In doing so, we highlight new crop improvement strategies for established and orphan crop systems.

## Supplementary Information

Below is the link to the electronic supplementary material.

Supplementary file1 (XLSX 90 kb)

## Data Availability

No datasets were generated or analysed during the current study.
